# Supplementary material for: Androgen receptor variant shows heterogeneous expression in prostate cancer according to differentiation stage
Source: Commun Biol. 2021 Jun 24;4:785. doi: 10.1038/s42003-021-02321-9 (PMC8225618; doi:10.1038/s42003-021-02321-9)
Supplement: Supplementary file 2 — Supplementary Information [file 42003_2021_2321_MOESM2_ESM.pdf]

Figure S1

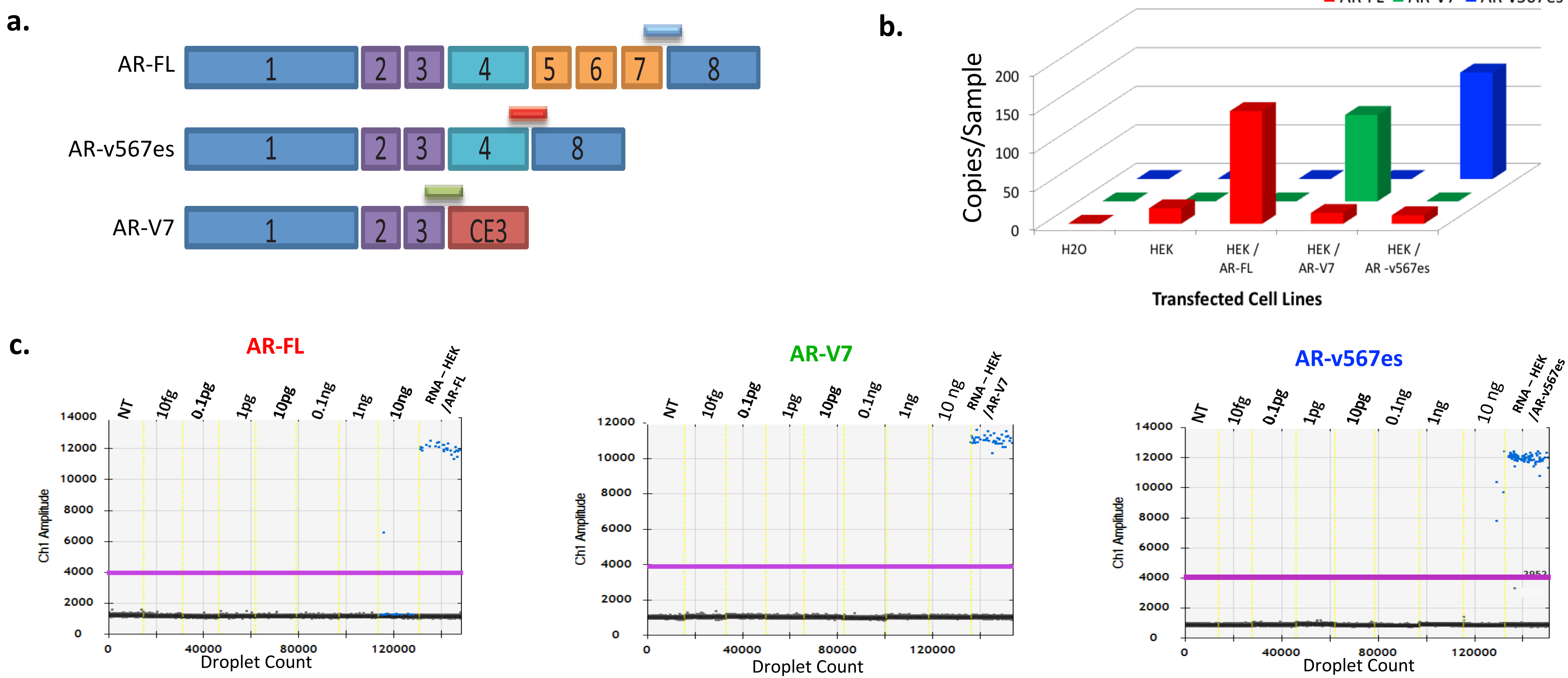

Figure S1. **Assay design and Specificity** **a**) Schematic of mRNA coding exons of AR-FL, AR-V7 and AR-v567es with primer location. **b**) AR-FL, AR-V7 and AR-v567es transcript detection (copies/sample) in HEK 293 cells transfected with empty vector (EV) or with each respective plasmid, as indicated, **c**) AR-FL, AR-V7 and AR-v567es transcript detection in genomic DNA of HEK 293 transfected with AR-FL, AR-V7 and AR-v567es respectively. RNA from HEK293 cells transfected with each respective transcript is included as positive control. Positive droplets above the threshold (magenta line) are shown in blue; negative droplets are shown in black. Each transcript detection is shown in the graph of fluorescence intensity (y-axis) *vs.* droplet number (x-axis).

Table S1.

Transcript	Type	Sequence
AR-FL	Forward	5’-AATCCCACATCCTGCTCAAG-3’
	Reverse	5’-GCAGCCTATTGCGAGAGAG-3’
	Probe	5’-ACCAGCTCACCAAGCTCCTGG-3’
	Fluorophore	FAM
AR-V7	Forward	5’-AGGGATGACTCTGGGAGAAA-3’
	Reverse	5’-AAAGGCTGACTTGCCTCATT-3’
	Probe	5’-TCCGGGTTGGCAATTGCAAGC-3’
	Fluorophore	FAM
AR^{v567es}	Forward	5’-CTTTGCAGCCTTGCTCTCTA-3’
	Reverse	5’-CTTGCCTGATTGCGAGAGAG-3’
	Probe	5’-ACACGTGGTCAAGTGGGCCA-3’
	Fluorophore	FAM

Table 1S. ddPCR primer and probe sequences of AR-FL, AR-V7 and AR-v567 transcripts

Figure S2

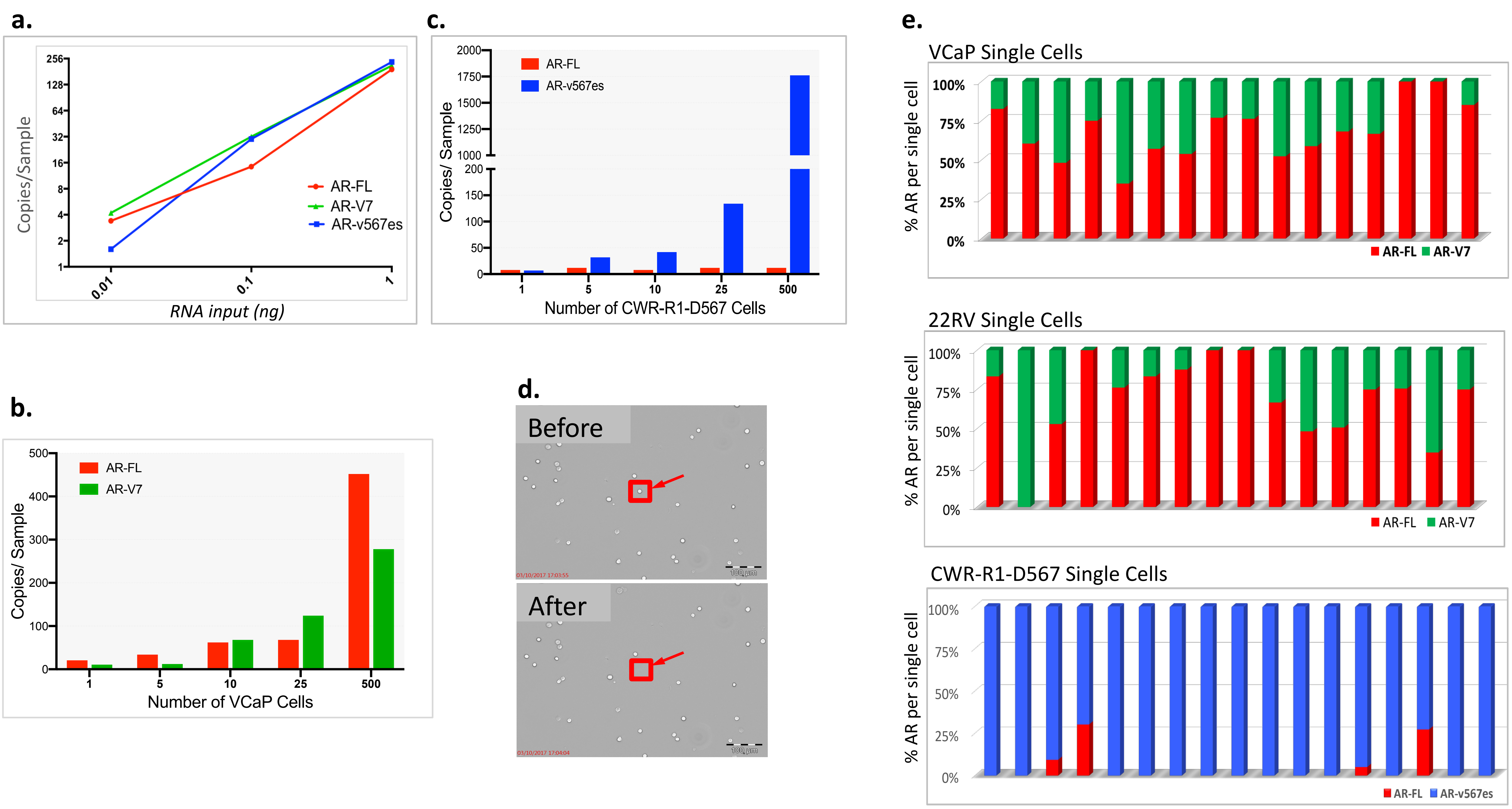
a.
Copies/Sample
256
128
64
32
16
8
4
2
1
0.01
0.1
1
RNA input (ng)
AR-FL
AR-V7
AR-v567es
b.
Copies/ Sample
500
400
300
200
100
0
AR-FL
AR-V7
1
5
10
25
500
Number of VCaP Cells
c.
Copies/ Sample
2000
1750
1500
1250
1000
200
150
100
50
0
AR-FL
AR-v567es
1
5
10
25
500
Number of CWR-R1-D567 Cells
d.
Before
After
100 µm
e.
VCaP Single Cells
22RV Single Cells
CWR-R1-D567 Single Cells
% AR per single cell
100%
75%
50%
25%
0%
AR-FL
AR-V7
AR-v567es

Figure S2. ddPCR Assay Sensitivity. **a**) cDNA was generated from RNA extracted from HEK293 cells transfected with each respective transcript and serially diluted as indicated. AR-FL, AR-V7 and AR-v567es transcript expression is shown as copies/sample. AR-FL, AR-V7 and AR-v567es transcript detection (copies/sample) in **b, c**) Pools of 1-500 VCaP (b) or CWR-R1-D567 (c) cells spiked spiked into 1 million of male healthy donor PBMCs. AR-FL, AR-V7 and AR-v567es transcripts are shown (copies/sample) **d**) Bright Field image of single cell before and after isolation by CellCelector **e**) AR-FL and AR-V7 transcript detection in single VCaP and 22Rv1 cells is shown in red and green colored bars, respectively. AR-FL and AR-v567es transcript detection in single CWR-R1-D567 cells is shown in red and blue colored bars, respectively. Expression of each transcript per single cell is displayed in 100% stacked column format. The relative amount of each AR-transcript is calculated as AR-X /(AR-FL+ AR-V7)*100

Table S2.

a.

Intra-assay Variability (n=5)		
Assay 1: AR-FL		
**Sample**	**Copies (SD)**	**CV%**
HEK293-NT	13.8 (±1.1)	8.4
HEK293-FL	1.5 (±0.05) x10^2	3.4
HEK293-V7	12.2(±1.1)	9.1
HEK293-v567es	13.8(±1.2)	8.4
Assay 2: AR-V7		
**Sample**	**Copies (SD)**	**CV%**
HEK293-NT	0 (0)	0
HEK293-FL	0 (0)	0
HEK293-V7	9.1 (±0.45) x10	5.0
HEK293-v567es	0 (0)	0
Assay 3: AR-v567es		
**Sample**	**Copies (SD)**	**CV%**
HEK293-NT	0 (0)	0
HEK293-FL	0 (0)	0
HEK293-V7	0 (0)	0
HEK293-v567es	1.67 (0.09) x10^2	5.4

b.

Inter-assay Variability (n=5)		
Assay 1: AR-FL		
**Sample**	**Copies (SD)**	**CV%**
HEK293-NT	12.9 (±0.79)	6.1
HEK293-FL	1.82 (±0.13) x10^2	7.2
HEK293-V7	12.6 (±0.9)	7.4
HEK293-v567es	11.9 (±0.8)	6.7
Assay 2: AR-V7		
**Sample**	**Copies (SD)**	**CV%**
HEK293-NT	0 (0)	0
HEK293-FL	0 (0)	0
HEK293-V7	9.6 (±0.2) x10	2.9
HEK293-v567es	0 (0)	0
Assay 3: AR-v567es		
**Sample**	**Copies (SD)**	**CV%**
HEK293-NT	0 (0)	0
HEK293-FL	0 (0)	0
HEK293-V7	0 (0)	0
HEK293-v567es	1.54 (0.1) x10^2	7.1

Table 2S. Intra-Assay (a) and Inter-Assay (b) Reproducibility: AR-FL, AR-V7 and AR-v567es transcript expression was assessed in 5 biological replicates. The table shows the raw data (copy number/sample) the SD and the % CV for all 5 replicates. Intra-assay results represent technical replicates of same batch of cells plated on 5 different plates; inter-assay results represent biological replicates where different batches of cells are processed on 5 different days.

Figure S3

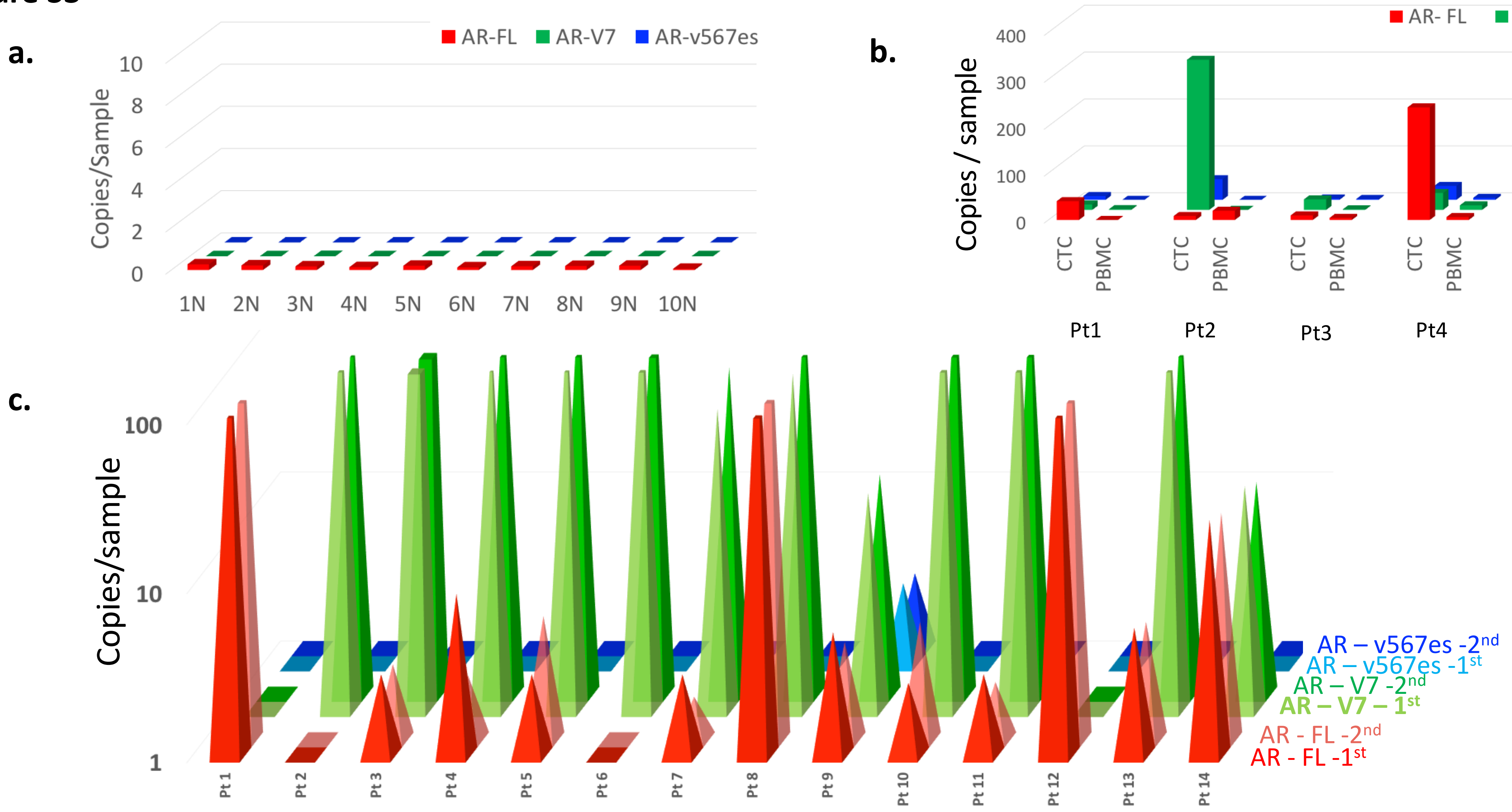

Figure S3. **Assay Validation and Reproducibility in Clinical samples a**) AR-FL, AR-V7 and AR-v567es expression (copies/sample) in PBMCs from male healthy donors (n=10), **b**) AR-FL (red), AR-V7 (green) and AR-v567es (blue) transcript detection in mCRPC patient CTCs (n=6) with matching PBMC fraction. **c**) Assay reproducibility in patient samples: RNA was extracted from mCRPC patient CTCs (n=14), split into two batches and processed by two independent operators on two different days. Transcript expression for AR-FL (red), AR-V7 (green) and AR-v567es (blue) is shown as copies/sample and displayed in solid (1st run) and gradient color (2nd run) for each patient and transcript.

Table S3

Characteristic	Value
No. of patients	29
Median age , years (range)	71 (55-90)
Median PSA, ng/mL (range)	17.6 (0.03-630.58)
Presence of bone metastases (%)	26 (89%)
Presence of visceral metastases (%)	5 (17%)
**Treatment history**	
No. of patients with prior therapy with ARSI (%)	14 (48%)
No. of patients with prior therapy with taxanes (%)	14 (48%)
No. of patients with prior therapy with ARSI and taxanes (%)	10 (34%)

Table S3. Summary of clinical characteristics
