# Supplementary material for: Androgen receptor variant shows heterogeneous expression in prostate cancer according to differentiation stage
Source: Commun Biol. 2021 Jun 24;4:785. doi: 10.1038/s42003-021-02321-9 (PMC8225618; doi:10.1038/s42003-021-02321-9)
Supplement: Supplementary file 3 — Description of Supplementary Files [file 42003_2021_2321_MOESM3_ESM.pdf]

Description of Additional Supplementary Files

File name: Supplementary Data 1
Description: Source data for main figures 1 - 3.

Figure 1: Excel sheet shows copy number of AR-FL, AR-V7 and AR-v567es transcripts in CTCs from each mCRPC patient.

Figure 2: Excel sheet tab labelled *Figure 2a* shows read counts of AR-FL and AR-V7 transcripts in TCGA and SU2C prostate cancer samples. Excel sheet tabs labelled *Figure 2b, 2c and 2d* shows read counts of AR-FL, AR-V7 and ARv567es in benign, primary PCA and mCRPC for each transcript respectively. These data were used to generate Figure 2.

Figure 3: Excel sheet shows copy number of AR-FL, AR-V7 and AR-v567es transcripts in $EpCAM^{pos}$ and $EpCAM^{neg}$ CTC pools in patients with mCRPC.
