# Supplementary material for: Androgen receptor variant shows heterogeneous expression in prostate cancer according to differentiation stage
Source: Commun Biol. 2021 Jun 24;4:785. doi: 10.1038/s42003-021-02321-9 (PMC8225618; doi:10.1038/s42003-021-02321-9)
Supplement: Supplementary file 5 — Reporting Summary [file 42003_2021_2321_MOESM5_ESM.pdf]

nature research

Corresponding author(s): COMMSBIO-20-0777A, Paraskevi Giannakakou, Ph.D.

Last updated by author(s): May 6, 2021

Reporting Summary

Nature Research wishes to improve the reproducibility of the work that we publish. This form provides structure for consistency and transparency in reporting. For further information on Nature Research policies, see our Editorial Policies and the Editorial Policy Checklist.

Statistics

For all statistical analyses, confirm that the following items are present in the figure legend, table legend, main text, or Methods section.

n/a	Confirmed	
☐	☒	The exact sample size (*n*) for each experimental group/condition, given as a discrete number and unit of measurement
☐	☒	A statement on whether measurements were taken from distinct samples or whether the same sample was measured repeatedly
☐	☒	The statistical test(s) used AND whether they are one- or two-sided<br>*Only common tests should be described solely by name; describe more complex techniques in the Methods section.*
☒	☐	A description of all covariates tested
☒	☐	A description of any assumptions or corrections, such as tests of normality and adjustment for multiple comparisons
☐	☒	A full description of the statistical parameters including central tendency (e.g. means) or other basic estimates (e.g. regression coefficient) AND variation (e.g. standard deviation) or associated estimates of uncertainty (e.g. confidence intervals)
☐	☒	For null hypothesis testing, the test statistic (e.g. *F*, *t*, *r*) with confidence intervals, effect sizes, degrees of freedom and *P* value noted<br>*Give P values as exact values whenever suitable.*
☒	☐	For Bayesian analysis, information on the choice of priors and Markov chain Monte Carlo settings
☒	☐	For hierarchical and complex designs, identification of the appropriate level for tests and full reporting of outcomes
☒	☐	Estimates of effect sizes (e.g. Cohen's *d*, Pearson's *r*), indicating how they were calculated

Our web collection on statistics for biologists contains articles on many of the points above.

Software and code

Policy information about availability of computer code

Data collection	The Cancer Genome Atlas (TCGA) raw data were directly downloaded from TCGA database, and Stand Up To Cancer (SU2C) raw data were directly downloaded from the database of Genotypes and Phenotypes (dbGaP).
Data analysis	Raw RNASeq short reads were trimmed using Trimmomatic (version 0.32) to eliminate low quality reads and aligned to human reference genome (version hg38) using STAR (2.6). After mapping, all splicing junction reads information were categorized in file_name.SJ.out.tab file. AR-FL expression was determined based on mapped reads across the junction between exon 7 and exon 8 of the AR gene. AR-V7 expression was determined based on mapped reads across the junction between exon 3 and cryptic exon 3 of the AR gene. AR-v567es expression was determined based on mapped reads across the junction between exon 4 and exon 8 of the AR gene.

For manuscripts utilizing custom algorithms or software that are central to the research but not yet described in published literature, software must be made available to editors and reviewers. We strongly encourage code deposition in a community repository (e.g. GitHub). See the Nature Research guidelines for submitting code & software for further information.

Data

Policy information about availability of data

All manuscripts must include a data availability statement. This statement should provide the following information, where applicable:

- Accession codes, unique identifiers, or web links for publicly available datasets
- A list of figures that have associated raw data
- A description of any restrictions on data availability

All bioinformatic's tools used in this manuscript were open source software, they are available publicly.

Field-specific reporting

Please select the one below that is the best fit for your research. If you are not sure, read the appropriate sections before making your selection.

☒ Life sciences ☐ Behavioural & social sciences ☐ Ecological, evolutionary & environmental sciences

For a reference copy of the document with all sections, see nature.com/documents/nr-reporting-summary-flat.pdf

Life sciences study design

All studies must disclose on these points even when the disclosure is negative.

Sample size	Peripheral blood were collected from patients with mCRPC (n=41), NEPC (n=2) and healthy male (n=10) subjects at Weill Cornell Medicine We also used RNA seq data that already have been deposited to The Cancer Genome Atlas database (TCGA),n=505 samples and from the Stand Up to Cancer (SU2C) datasets that have deposited at the database of Genotypes and Phenotypes (dbGaP), n=98 patient samples . For the Nanostring analysis, the data were obtained from patients at our Institution with benign prostate tissue (n=49), localized prostate cancer (n=89) and CRPC (n=39).
Data exclusions	No data were excluded from the analysis.
Replication	We tested the intra- and inter-assay reproducibility by assessing the expression of AR-FL, AR-V7 and AR-v567es transcripts in 5 biological replicates. For the intra-assay reproducibility we performed 5 technical replicates of same batch of cells plates on 5 different plates; for the inter-assay reproducibility we performed biological replicates of different batches of cells processed on 5 different days.
Randomization	No randomization was performed.
Blinding	Investigators were blinded to patient clinical outcomes

Reporting for specific materials, systems and methods

We require information from authors about some types of materials, experimental systems and methods used in many studies. Here, indicate whether each material, system or method listed is relevant to your study. If you are not sure if a list item applies to your research, read the appropriate section before selecting a response.

Materials & experimental systems

n/a	Involved in the study	
☐	☒	Antibodies
☐	☒	Eukaryotic cell lines
☒	☐	Palaeontology and archaeology
☒	☐	Animals and other organisms
☐	☒	Human research participants
☐	☒	Clinical data
☒	☐	Dual use research of concern

Methods

n/a	Involved in the study	
☒	☐	ChIP-seq
☒	☐	Flow cytometry
☒	☐	MRI-based neuroimaging

Antibodies

Antibodies used	RosetteSep™ Human CD45 Depletion cocktail, STEMCELL Technologies, Catalog # 15162; EpCAM mAb, clone D4K8R, Cell Signaling, Cat.# 36746; Pacific Blue™ anti-human CD45, clone H130, Biolegend, Cat. # 982306;
Validation	We validated antibody specificity by using positive and negative controls for the target antigen, and all other controls appropriate for immunofluorescence microscopy such as no template control, secondary antibody only control.

Eukaryotic cell lines

Policy information about cell lines

Cell line source(s)	22Rv1 (Cat # CRL-2505) and VCaP (Cat # CRL-2876) cells were obtained from ATCC; CWR-R1-D567 cells were kindly gifted to us from Dr. Scott Dehm (University of Minnesota)
Authentication	ATCC authenticates human cancer cell lines using short tandem repeat analysis
Mycoplasma contamination	All our cell lines tested negative for mycoplasma contamination. We regularly check all our cell lines every month to make sure they are free of mycoplasma.

Commonly misidentified lines (See ICLAC register)	N/A

Human research participants

Policy information about studies involving human research participants

Population characteristics	The median age of patient population for the AR-V assay was 71 years, (range 55-90). All human subjects collected were from male patients or healthy volunteers.
Recruitment	Patients with metastatic prostate cancer or NEPC were recruited from routine clinical operations receiving standard-of-care treatment.
Ethics oversight	Weill Cornell Institutional Review Board Protocol #0707009283-01

Note that full information on the approval of the study protocol must also be provided in the manuscript.

Clinical data

Policy information about clinical studies

All manuscripts should comply with the ICMJEguidelines for publication of clinical research and a completedCONSORT checklist must be included with all submissions.

Clinical trial registration	NA
Study protocol	NA
Data collection	NA
Outcomes	NA
